# Reliability, validity and responsiveness of a Norwegian version of the Chronic Sinusitis Survey

**DOI:** 10.1186/1472-6815-6-9

**Published:** 2006-05-05

**Authors:** Knut Stavem, Edna Røssberg, Pål G Larsson

**Affiliations:** 1 Medical Department, Akershus University Hospital, Lørenskog, Norway; 2 Helse Øst Health Services Research Centre, Lørenskog, Norway; 3 Balder-Klinikken, Oslo, Norway; 4 Department of Clinical Neurophysiology, The National Centre for Epilepsy, Sandvika, Norway

## Abstract

**Background:**

The Chronic Sinusitis Survey (CSS) is a valid, disease-specific questionnaire for assessing health status and treatment effectiveness in chronic rhinosinusitis. In the present study, we developed a Norwegian version of the CSS and assessed its psychometric properties.

**Methods:**

In the pooled data set of 65 patients from a trial of treatment for chronic sinusitis with long-standing symptoms and signs of sinusitis on computed tomography (CT), we assessed the reliability, validity and responsiveness of the CSS.

**Results:**

Test-retest reliability of the two CSS scales and the total scale ranged 0.87–0.92, while internal consistency reliability ranged 0.31–0.55. CSS subscale scores were associated with other items on sinusitis symptoms, and with the Mental health and Bodily pain scale of the SF-36. There was little association of the CSS scale scores with sinus CT findings. The patients with chronic sinusitis had worse scores on all three CSS scales than a healthy reference population (n = 42) (p < 0.001). The CSS sinus symptoms subscale and the total scale were sensitive to improvement in global symptoms during 12 weeks.

**Conclusion:**

The Norwegian version of the CSS had acceptable test-retest reliability, but lower internal consistency reliability than the accepted standard criteria. The results support the construct validity of the measure and the sinusitis symptoms subscale and the total scales were responsive to change. This supports the use of the questionnaire in interventions for chronic sinusitis, but points at problems with the internal consistency reliability.

## Background

Sinusitis is a common condition that causes frequent physician visits. In the United States in 1999–2000, chronic sinusitis was accountable for more than 13 million ambulatory care visits, or 1.3 % of all visits [1]. The symptoms of chronic sinusitis are not easy to quantify [2] and show little association with computed tomography (CT) findings [3]. Postoperatively, imaging is also unreliable because of postoperative changes and scarring [4].

Health-related quality of life (HRQoL) has recently gained increased awareness as an outcome measure for interventions in chronic sinusitis [2,5-11]. Some studies of interventions in chronic sinusitis have used generic HRQoL measures, which are developed for use in a wide range of conditions and typically include aspects of physical, emotional and social health or functioning, such as the Short Form 36 (SF-36) [12]. To increase the sensitivity to change, disease-specific measures have been developed to assess the impairment of chronic sinusitis and the effects of interventions. A review reported that only three instrument for outcome in chronic sinusitis had acceptable performance characteristics with documented reliability, validity and responsiveness [5,13-15], however new instruments have later been introduced [16].

The Chronic Sinusitis Survey (CSS) is a valid, disease-specific questionnaire for assessing health status and treatment effectiveness in chronic rhinosinusitis [2,5,10]. In the present study, we developed a Norwegian version of the CSS and assessed its psychometric properties in the pooled sample of patients in an intervention for chronic sinusitis [17].

## Methods

### Subjects and study design

We included patients above 17 years of age with sinusitis symptoms for more than three months and sinus swelling, fluid retention, or opacification on CT. Patients with polypous sinusitis or pansinusitis, pregnancy, previous acupuncture treatment, previous surgery for chronic sinusitis, or recent medication use that could influence the results of the study were excluded [17]. We evaluated more than 500 patients with sinusitis for eligibility to the study. In total, 65 patients were included from August 1996 to December 2000. Patients were initially recruited from the clinical practice of one otorhino-laryngologist and later also through advertising in local newspapers and a magazine.

One otorhino-laryngologist examined and included all patients. He allocated them to one of three groups according to a six-block randomisation algorithm. The major reasons for exclusion estimated post hoc by the otorhino-laryngologist were: normal CT (30%), heavy allergies (20%), refused conventional medical therapy (10%), trigeminal neuralgia (10%) and did not want a CT-scan (5%).

The patients had one of three treatments: conventional medical therapy with antibiotics and local congestants, traditional Chinese acupuncture, or minimal acupuncture at non-acupoints. No treatments were given during the Norwegian allergy season (February – September), because some of the patients were expected to have seasonal allergies [17]. For the purpose of this validation study, we pooled the three study arms in the analysis.

### Health status assessment

We assessed HRQoL at baseline, after 12 weeks and 13 weeks with several instruments. The purpose of the 13-week assessment was to assess test-retest reliability of the questionnaires, by comparison with the 12-week assessment.

#### Chronic sinusitis survey (CSS)

The CSS is a 6-item duration-based, sinus-specific questionnaire with a symptom and a medication subscale. It is developed at the Massachusetts Eye and Ear Infirmary [2,5,10] and also exists in Chinese and Turkish versions [18,19]. We obtained permission to use the CSS and translated the questionnaire to Norwegian according to a recommended procedure [20]. First, two individuals translated the CSS into Norwegian independently. They then met and discussed with a third person, agreeing on a consensus version. Later, this consensus version was backtranslated into English by an American fluent in Norwegian. Comparison of the backtranslation with the original English version revealed little discrepancies, and the questionnaires were considered conceptually and linguistically equivalent. The Norwegian version of the CSS is enclosed ' [see Additional file 1]'.

#### Short Form 36 (SF-36)

The general health status questionnaire SF-36 assesses eight dimensions of health status including physical functioning, role limitations due to physical problems, bodily pain, general health, vitality, social functioning, role limitations due to emotional problems and mental health [12,21]. The scales were scored from 0 (lowest level of functioning) to 100 (highest level of functioning). The SF-36 has been extensively validated in general populations [12,21], and in many diseases including subjects with chronic rhinosinusitis [9,10,15,22]. We used the Norwegian standard SF-36 version 1.2 [23]. Additionally, we scored the physical component summary (PCS) and mental component summary (MCS) scales [24]. These two scales were scored and transformed for comparison with a U.S. general population with mean 50 and SD 10.

#### Sinus computed tomography

At baseline and 4–6 weeks later the patients had sinus CT scans. An otorhino-laryngologist assessed soft tissue swelling in millimetres and signs of fluid retention and opacification on the CT-scans [17].

### Sinusitis symptom assessment

The patients reported on six symptoms of chronic sinusitis using a self-administered questionnaire [17]: (1) mucus production, (2) maxillary headache, (3) stuffed nose, (4) frontal headache, (5) ability to smell, and (5) feeling of illness. The first four items were scored on an ordinal scale with the response alternatives none, little, some, much, very much (and recoded on a 0–4 scale). The fifth item had the response alternatives none, little, some, close to normal, normal (recoded on a 4-0 scale). The sixth item had the response alternatives very ill, ill, a little ill, healthy (recoded 3-0). The recoded six symptom scores were summed to give an aggregate value representing a "sinusitis symptom score", with a score ranging from 0 (minimal symptoms) to 23 (maximal symptoms) [17].

### Healthy comparison group

To have a healthy comparison group, with which we could compare the CSS scores, we used a convenience sample of hospital personnel (n = 42). These subjects only responded to the CSS and items on age and sex.

### Statistical analysis

Descriptive statistics are presented with means and SDs, or percentages. Group characteristics were compared using the t-test or χ^2 ^test.

Internal consistency reliability of the scales was assessed using Cronbach's α [25]. Test-retest reliability of the aggregate scales was assessed with an intraclass correlation coefficient (ICC), using the average of raters in a two-way mixed model with an absolute agreement definition. To minimize the subjects' recall for the previous answers, the test-retest was done using individual patient scores 12 and 13 weeks after randomization. We excluded assessments more than 21 days apart. The time between the assessments was median 7 days (range 6 to 21).

Construct validity of the scales of the CSS was assessed by correlations with: (1) corresponding items of the SF-36, and (2) the items on the sinusitis symptom scale. We used Spearman's rank correlations because of the ordinal scale on sinusitis symptoms items. A finding of higher correlations between items measuring related phenomena than between non-corresponding items would support construct validity.

The discriminant validity of the CSS and the SF-36 was evaluated by the capacity of the subscales to differentiate between two groups with expected differences in health status. For this purpose, we used grading of CT sinus soft tissue swelling (sum of six measures of sinus soft tissue swelling) in millimeters, dividing the patients into two groups according to score below or above the median of 12 mm. In this comparison, we adjusted for age using analysis of variance. We similarly compared scores between two groups divided according to the median of the overall symptom score (≤ 9 vs. >9).

Finally, we compared CSS scores in the total sample of patients with chronic sinusitis with the healthy comparison group of hospital personnel, using the t-test for independent samples.

Responsiveness was assessed in the pooled sample using change in the overall symptom score as an indicator of global change. The SD of the baseline overall symptom score was 3.1. We used a change of 2 units on the (equivalent to 0.65 SD) to denote a meaningful change. We had no empirical evidence for this, however previous reports have suggested that minimally clinical important changes frequently are about 0.5 SD [26], hence our choice is in accordance with this. We report responsiveness as standardized response mean (SRM) (mean change/SD of change) and effect size (mean change/SD at baseline) [27]. Because patients in one of the treatment arms were given antibiotics and other medication as part of the protocol, we excluded patients in this treatment arm from the analysis of responsiveness of the CSS Medication usage and Total scales. These scales are influenced directly by medication use. Instead, we would expect this group to report deterioration in score on the CSS Medication usage subscale.

We chose a significance level of 5%. For statistical analysis, we used Stata version 8.2 (Stata Corp., College Station, TX) and SPSS version 12.0 (SPSS Inc, Chicago, IL). The Regional Committee for Medical Research Ethics and The Norwegian Data Inspectorate approved the study.

## Results

The mean age of the participants was 43 years, 51% were women and 35% were current smokers. On baseline sinus CT, 17 patients had opacification, 2 had fluid retention, and 57 had sinus soft tissue swelling. There was no difference in age or sex between the chronic sinusitis group and the healthy comparison group (table 1).

**Table 1:** Baseline subject characteristics, mean (SD)

	Chronic sinusitis	Healthy controls	p
N	65	42	
Women, number (%)	33 (51)	25 (60)	0.38
Age (years)	43.0 (13.8)	42.8 (12.1)	0.95
Duration of chronic sinusitis (years)	9.5 (10.7)	-	-
Known allergy, number (%)	12 (19)	-	-
Current smokers, number (%)	23 (35)	-	-
Sinus soft tissue swelling in mm*	13.3 (10.6)	-	-

*Sum of 2 maxillary, 2 frontal, ethmoidal and sphenoidal soft tissue swelling measurements, n = 64

Completers, who responded to the sinusitis symptoms scale of the CSS at baseline and after 12 weeks (n = 47), tended to be somewhat older, had suffered from chronic sinusitis longer, and had better baseline CSS and SF-36 scores on all subscales than dropouts (n = 18) after 12 weeks, although only the differences on the General health (p = 0.03) and the Social functioning scales (p = 0.04) of the SF-36 were statistically significant.

At baseline the mean CSS sinus symptoms score was 40 (SD 26) and the medication usage score 83 (SD 18) (table 2). On the sinus symptoms scale, 13% of respondents scored the lowest possible value and on the medication usage scale 32% of respondents scored the highest possible value, the latter indicting an apparent ceiling effect. On the SF-36 scales, there were signs of a floor effect on the Role-physical and Role-emotional scales, and ceiling effects on the Physical functioning, Role-physical, Bodily pain, Social functioning and Role-emotional scales (table 2).

**Table 2:** Baseline health-related quality of life scores and reliability assessments

	N	Number of items	Range	Mean (SD)	%Floor	%Ceiling	Internal consistency reliability*	Test-retest reliability**
*Chronic sinusitis survey (0–100 scale)*								
Sinus symptoms	63	3	0–100	40 (26)	13	2	0.55	0.92
Medication usage	62	3	17–100	83 (18)	0	32	0.31	0.87
Total	61	6	29–92	62 (16)	0	0	0.39	0.94
*SF-36 (0–100 scale)*								
Physical functioning	63	10	10–100	82 (20)	0	16	0.91	0.93
Role – physical	64	4	0–100	54 (41)	25	33	0.83	0.86
Bodily pain	64	2	0–100	60 (23)	2	13	0.86	0.88
General health	64	5	15–100	59 (23)	2	2	0.76	0.94
Vitality	64	4	0–85	47 (23)	5	0	0.87	0.90
Social functioning	64	2	13–100	69 (26)	0	23	0.81	0.84
Role – emotional	62	3	0–100	66 (38)	16	45	0.72	0.76
Mental health	64	5	16–100	74 (17)	0	2	0.82	0.89
Physical component summary***	61	35	21–61	44 (9)	-	-	-	0.94
Mental component summary***	61	35	23–59	46 (11)	-	-	-	0.90

%Floor = percentage of patients scoring lowest possible value, %Ceiling = percentage of patients scoring highest possible value.

* Cronbach's alpha, ** Intraclass correlation coefficient, N = 38–41, *** Standardized for comparison with an American general population with mean 50, SD 10.

Internal consistency relibility, as assessed with Cronbach's α, ranged 0.31 to 0.55 for the CSS scales and 0.72 to 0.91 for the eight SF-36 scales (table 2). Test retest reliability ranged 0.87 to 0.94 for the CSS scales and 0.76 to 0.94 for the SF-36 scales (table 2).

The multitrait-multimethod matrix showed that the correlations between the CSS Sinus symptoms subscale had the highest correlations with other symptom items or scales, such as those on mucus production (-0.38), stuffed nose (-0.45), general feeling of illness (-0.43) and the aggregated sum of symptoms score (-0.45) and the SF-36 scale Bodily Pain (0.36) (table 3). In contrast there was little association of the medication usage scale with the SF-36 scales and the symptom items. The CSS total scale, which represents an average of the two subscales, showed high correlation with the Role-emotional scale of the SF-36 (0.73) and moderate correlation with the Bodily pain scale (0.41) of the SF-36 and the feeling of illness item (-0.44) and Sum of symptoms scale (-0.41).

**Table 3:** Spearman's rank correlation coefficients between baseline scores on scales of the Chronic sinusitis survey, SF-36, and items of a sinusitis symptoms scale (n = 60–63)

	Chronic sinusitis survey scale		
	Sinus symptoms	Medication usage	Total

*SF-36 scale*			
Physical functioning	-0.13	0.17	-0.02
Role – physical	-0.16	0.1	0.2
Bodily pain	0.36*	0.15	0.41**
General health	-0.08	0.14	0.03
Vitality	0.08	-0.07	0.01
Social functioning	0.05	0.1	0.12
Role – emotional	0.08	-0.04	0.73
Mental health	0.21	-0.03	0.17
Physical component summary	0.08	0.19	0.19
Mental component summary	0.10	-0.06	0.08
*Sinusitis symptoms*			
Mucus production	-0.38**	0.18	-0.25
Stuffed nose	-0.45**	0.14	-0.29*
Frontal headache	-0.32**	0.04	-0.26*
Maxillary headache	-0.11	-0.1	-0.19
Ability to smell	0.02	-0.13	-0.09
Feeling of illness	-0.43**	-0.08	-0.44**
Sum of symptoms	-0.45**	-0.02	-0.41**

* ≤ 0.05, ** ≤ 0.01

The CSS and SF-36 scales were not able to discriminate between the group of patients with aggregate sinus soft tissue swelling ≤ 12 mm versus >12 mm (table 4). In contrast, the CSS sinus symptom and total scales, and the SF-36 Bodily Pain and Mental health scales discriminated between patients divided into groups according to overall symptom scale score above or below the median (table 4). When comparing CSS scores in the total sample of patients with chronic sinusitis with the healthy comparison group, there was a marked difference in scores on the two subscales and the total score (table 5).

**Table 4:** Age-adjusted quality of life scores according to baseline CT scores among those with sinus soft tissue swelling (n = 45) and overall symptom score (groups defined with the median as cut-off), mean (SE)

	CT soft tissue swelling				Overall symptom score		
	None	≤ 12 mm	>12 mm	p*	≤ 9	>9	p

n	10	23	22		37–39	24	
*Chronic sinusitis survey (0–100 scale)*							
Sinus symptoms	49 (9)	40 (6)	38 (5)	0.80	48 (4)	28 (5)	0.005
Medication usage	82 (6)	84 (4)	83 (3)	0.86	84 (3)	82 (4)	0.71
Total	65 (5)	63 (4)	60 (3)	0.56	66 (3)	55 (3)	0.01
*Short Form 36 (0–100 scale)*							
Physical functioning	81 (7)	84 (4)	82 (4)	0.70	85 (3)	78 (4)	0.16
Role – physical	61 (13)	47 (9)	56 (7)	0.42	57 (6)	48 (8)	0.43
Bodily pain	58 (7)	63 (5)	57 (4)	0.34	66 (3)	48 (4)	0.002
General health	61 (7)	61 (5)	56 (4)	0.50	58 (4)	59 (5)	0.83
Vitality	52 (7)	47 (5)	46 (4)	0.87	49 (4)	45 (5)	0.50
Social functioning	69 (8)	71 (6)	68 (5)	0.68	72 (4)	65 (5)	0.28
Role – emotional	69 (12)	71 (8)	60 (7)	0.31	70 (6)	58 (8)	0.28
Mental health	73 (5)	77 (4)	72 (3)	0.29	77 (3)	69 (3)	0.05
PCS	45 (3)	43 (2)	44 (2)	0.81	45 (2)	42 (2)	0.22
MCS	46 (4)	48 (2)	45 (2)	0.33	47 (2)	44 (2)	0.28

* comparison of ≤ 12 mm group vs. >12 mm

**Table 5:** Chronic sinusitis survey scores (0–100 scale) for patients with chronicsinusitis compared with a healthy reference population, mean (SD)

	Chronic sinusitis	Healthy controls	p
n	61–63	42	
Sinus symptoms	40 (26)	87 (19)	<0.001
Medication usage	83 (18)	98 (6)	<0.001
Total	61 (16)	92 (11)	<0.001

Of the 47 patients that completed the study, 22 reported improved overall symptom score (change < -2 units), 19 were unchanged (change of -2 to 2 units), and 6 were worse (change > 2 units). The worse group was considered too small for further analysis. The responsiveness indices in general were larger in the improved group than in the unchanged group and in the right direction for all indices (table 6). The CSS scales sinusitis symptoms and total (SRM of 0.66–1.03, ES 0.69 to 1.29) and the SF-36 scales Bodily pain, Social functioning, and Physical component summary (SRM 0.65–0.84; ES 0.65 to 0.79) were the scales most responsive to improvement (table 6). The medication usage scale showed little sensitivity to change.

**Table 6:** Change in quality of life scores according to changes in overall symptom score from baseline to week 12

	Unchanged					Improved				
	N	Mean change 0 to 12 weeks	SD of change within group	SRM	Effect size	N	Mean change 0 to 12 weeks	SD of change within group	SRM	Effect size

*Chronic sinusitis survey*										
Sinus symptoms	18	2	25	0.09	0.08	20	33	32	1.02	1.29
Medication usage*	15	2	22	0.08	0.08	11	5	24	0.20	0.25
Total*	15	5	15	0.31	0.28	10	13	21	0.66	0.69
*Short Form 36*										
Physical functioning	19	-2	9	-0.17	-0.11	22	10	20	0.49	0.39
Role – physical	19	-3	33	-0.08	-0.07	22	20	42	0.47	0.51
Bodily pain	19	3	16	0.16	0.10	22	16	25	0.65	0.74
General health	19	-5	14	-0.38	-0.23	22	12	21	0.55	0.47
Vitality	19	5	15	0.31	0.22	22	9	26	0.33	0.36
Social functioning	19	1	16	0.08	0.06	22	17	20	0.84	0.65
Role – emotional	19	4	27	0.13	0.12	22	9	37	0.24	0.24
Mental health	19	-0.3	15	-0.02	-0.02	22	5	17	0.28	0.23
Physical component summary	19	-1	5	-0.18	-0.12	22	6	9	0.74	0.79
Mental component summary	19	1	6	0.23	0.18	22	3	11	0.28	0.25

SRM = standardized response mean; *patients receiving conventional medical treatment with medication were excluded from this analysis (n = 14).

In the conventional group with medication as part of the intervention, the unchanged symptom group reported SRM and ES of -0.29 (n = 4). In the improved group SRM was -0.40 and ES -0.64 (n = 10), in accordance with increased use of medication during the study than before.

## Discussion

In this study we have documented translation of the CSS into Norwegian and assessed aspects of its reliability, validity and responsiveness in patients with CT-verified chronic sinusitis. The internal consistency reliability of the CSS scales was moderate to fair, and lower than the level of 0.70 usually considered acceptable for group use [28].

The internal consistency reliability in the present study was lower than previously reported for the CSS scales in some studies [5,18], for some scales of the RhinoQOL [6,16] and some other instruments [14,15,29,30]. However, the internal consistency reliability was at the level of the CSS in another study [6] and some other RhinoQOL scales [6,18]. In contrast all, eight SF-36 scales in the present study had internal consistency reliability <0.70. The problems with internal consistency of the CSS could be related to the low number of CSS items, as internal consistency normally increases with increasing number of items. Hence, there is a trade-off between ease of administration and internal consistency.

The test-retest reliability in the present study was substantial for all CSS and SF-36 scales, as previously reported for the CSS [5,18] and higher than reported for some other disease-specific instruments [6,29]. However, these comparisons should be interpreted carefully, because of differences in samples and assessment methods.

Assessment of cross-sectional validity in the present study showed that the CSS total scale was moderately associated with the Bodily pain scale of the SF-36, at the level previously reported [5]. The association of the CSS total with the Role-emotional scale of the SF-36 was higher than previously reported [5]. Further, there was marked difference between CSS subscale and total scores among patients with chronic sinusitis and the healthy comparison group. These findings and the associations with symptom scores were in line with expectations and give support to construct validity of the CSS [31]. In contrast, when relating the CSS and SF-36 scale scores to the CT findings, there was little association. This finding supports previous findings that there is little association between symptoms and CT based severity staging in chronic sinusitis [32-34]. The results from the comparison of CSS scores with healthy controls in the present study are in line with previous similar comparisons with general population values [2,35].

Some subscales of both the CSS and the SF-36 were sensitive to change in this pooled sample of patients receiving three different interventions, in accordance with previous reports of surgical interventions [2,6,10] The ES for the responsive scales ranged from medium to large, using the nomenclature of Cohen, where an ES of 0.2 is considered small, 0.5 medium, and 0.8 represents a large ES [36]. In the present study, the sinusitis symptoms subscale of the CSS was more responsive than the medication use subscale, as previously reported [6]. The medication use subscale of the CSS is not feasible for use with interventions using pharmaceuticals, as in one of the arms of the present study; however, we think this subscale is more justified in surgical interventions.

Compared with other disease-specific questionnaires for use in chronic sinusitis, the CSS is short, easy to use, has documented validity, and scores are available for healthy populations. In the present study, we did not compare the questionnaire with other disease-specific instruments. However, we think the CSS is a feasible disease-specific instrument for use in many interventions, and more documentation exists for this instrument than for other sinus-specific HRQoL instruments.

Some weaknesses of our study should be mentioned. The sample size was small, hence reducing the power of the study. Periodicity or seasonality of symptoms or environmental factors might influence our measured outcomes. In lack of a gold standard, we compared scores in chronic sinusitis with those in a healthy convenience sample and assessed cross-sectional associations with CT findings and a symptom score that we had developed for this study. However, this scale has not been subject to the same rigorous testing as the HRQoL measures. We based its use in the present study on an assumption of face validity of the items. We also did not use a standardized and validated CT staging system, but the lack of association with our system was in accordance with previous reports [32-34]. Responsiveness was assessed using change in overall symptoms as benchmark, which we thought was the best estimate of global outcome that we could find.

Because we only included patients with CT-verified sinusitis, we included a small proportion of all patients presenting symptoms of chronic sinusitis. Hence, one should be careful extrapolating the results of the study beyond patients with CT-verified sinus soft tissue swelling.

## Conclusion

We have documented the translation of the CSS into Norwegian and shown that this version of the CSS had substantial test-retest reliability, but there were problems with the internal consistency reliability. This suggests that the two three-point scales were not homogeneous. The cross-sectional associations give support to validity of the scales in chronic sinusitis. Finally, scales of the CSS and the SF-36 were responsive in this pooled population receiving three different interventions.

## Abbreviations

CSS = chronic sinusitis survey; ICC = intraclass correlation coefficient; CT = computed tomography; SF-36 = short form 36; SRM = standardized response mean; ES = effect size

## Competing interests

The author(s) declare that they have no competing interest

## Authors' contributions

ER initiated the study, wrote the protocol, provided funding, organized the data collection and critically reviewed the paper. KS participated in study planning, writing of the protocol, performed the statistical analysis, drafted the paper and wrote the final version. PGL participated in study planning, writing of the protocol, data management and critically reviewed and commented on the paper. All authors approved the final version.

## Pre-publication history

The pre-publication history for this paper can be accessed here:



## Supplementary Material

Additional file 1 The Norwegian version of the Chronic Sinusitis Survey – Duration based. The Norwegian translation of the 'Chronic Sinusitis Survey – Duration based' questionnaire Click here for file
