# Supplementary material for: Reliability, validity and responsiveness of a Norwegian version of the Chronic Sinusitis Survey
Source: BMC Ear Nose Throat Disord. 2006 May 5;6:9. doi: 10.1186/1472-6815-6-9 (PMC1468424; doi:10.1186/1472-6815-6-9)
Supplement: Additional file 1 — The Norwegian version of the Chronic Sinusitis Survey – Duration based. The Norwegian translation of the 'Chronic Sinusitis Survey – Duration based' questionnaire [file 1472-6815-6-9-S1.doc]

**The Norwegian version of the Chronic Sinusitis Survey – Duration based**

Svar på hvert spørsmål ved å sette en ring rundt det tallet som passer. Hvis du er usikker

på hvordan du skal svare på et spørsmål, vennligst angi det svaret som passer best.

1. I løpet av de siste *8 ukene*, i hvor mange *uker* har du hatt: (sett en ring rundt ett svar

på hver linje)

a. Hodepine , smerte eller press i ansiktet fra bihulene

0 uker 1-2 uker 3-4 uker 5-6 uker 7-8 uker

b. Renning fra nesen eller renning fra nesen bakover i svelget

0 uker 1-2 uker 3-4 uker 5-6 uker 7-8 uker

c. Nesetetthet eller vansker med å puste gjennom nesen

0 uker 1-2 uker 3-4 uker 5-6 uker 7-8 uker

2. I løpet av de siste *8 ukene*, i hvor mange *uker* har du tatt: (sett en ring rundt ett svar

på hver linje)

a. Antibiotika

0 uker 1-2 uker 3-4 uker 5-6 uker 7-8 uker

b. Nesespray forordnet av din lege

0 uker 1-2 uker 3-4 uker 5-6 uker 7-8 uker

c. Medisiner for bihulene i tablettform (som antihistaminer eller

slimhinneavsvellende midler)

0 uker 1-2 uker 3-4 uker 5-6 uker 7-8 uker
